# The effectiveness of attentional bias modification for substance use disorder symptoms in adults: a systematic review

**DOI:** 10.1186/s13643-018-0822-6

**Published:** 2018-10-13

**Authors:** Janika Heitmann, Elise C. Bennik, Madelon E. van Hemel-Ruiter, Peter J. de Jong

**Affiliations:** 1 Verslavingszorg Noord Nederland, Groningen, The Netherlands; 2 Department of Clinical Psychology and Experimental Psychopathology, University of Groningen, Groningen, The Netherlands

**Keywords:** Attentional bias, Attentional bias modification, Cognitive bias modification, Addiction, Substance use disorder, Alcohol, Nicotine, Opiate, Systematic review

## Abstract

**Background:**

Attentional bias modification (ABM) interventions have been developed to address addiction by reducing attentional bias for substance-related cues. This study provides a systematic review of the effectiveness of ABM interventions in decreasing symptoms of addictive behaviour, taking baseline levels of attentional bias and changes in attentional bias into account.

**Methods:**

We included randomised and non-randomised studies that investigated the effectiveness of ABM interventions in heavy-using adults and treatment-seeking individuals with symptoms of substance use disorder to manipulate attentional bias and to reduce substance use-related symptoms. We searched for relevant English peer-reviewed articles without any restriction for the year of publication using PsycINFO, PubMed, and ISI Web in August 2016. Study quality was assessed regarding reporting, external validity, internal validity, and power of the study.

**Results:**

Eighteen studies were included: nine studies reported on ABM intervention effects in alcohol use, six studies on nicotine use, and three studies on opiate use. The included studies differed with regard to type of ABM intervention (modified dot probe task *n* = 14; Alcohol Attention Control Training Programme *n* = 4), outcome measures, amount and length of provided sessions, and context (clinic versus laboratory versus home environment). The study quality mostly ranged from low average to high average (one study scored below the quality cut-off). Ten studies reported significant changes of symptoms of addictive behaviour, whereas eight studies found no effect of ABM interventions on symptoms. However, when restricted to multi-session ABM intervention studies, eight out of ten studies found effects on symptoms of addiction. Surprisingly, these effects on symptoms of addictive behaviour showed no straightforward relationship with baseline attentional bias and its change from baseline to post-test.

**Conclusions:**

Despite a number of negative findings and the diversity of studies, multi-session ABM interventions, especially in the case of alcohol and when the Alcohol Attention Control Training Programme was used, appear to have positive effects on symptoms of addictive behaviour. However, more rigorous well-powered future research in clinical samples is needed before firm conclusions regarding the effectiveness of ABM interventions can be drawn.

**Systematic review registration:**

Registration number PROSPERO: CRD42016046823

**Electronic supplementary material:**

The online version of this article (10.1186/s13643-018-0822-6) contains supplementary material, which is available to authorized users.

## Background

The severity of problems relating alcohol and drug use [1], and the high lifetime prevalence of addiction [2] stress the importance of making effective treatments easily available for individuals experiencing problems with the use of addictive substances. Overall, evidence-based psychosocial treatments, such as cognitive behavioural therapy (CBT), contingency management (CM), and relapse prevention, have been found to be effective in short term [3, 4]. However, approximately half of the people treated for any substance use disorder relapse within the first year after treatment [5]. This might indicate that these interventions do not address all crucial components maintaining addiction.

Current dual process models of addiction point to the relevance of differentiating between more explicit and more implicit processes guiding addictive behaviour. Both processes have been proposed to contribute to the development and persistence of addiction [6, 7]. One of the implicit processes that have been identified as a potentially important process in addiction is attentional bias. Attentional bias has been defined as the tendency to implicitly focus on and keep attention on substance-relevant cues in the environment [8, 9], such as the pub on the other side of the street. Given that treatments, such as CBT and CM, mainly focus on explicit decision-making processes and have been found to be insufficiently effective in the adaptation of implicit processes like attentional bias [10], new interventions have been developed to directly modify these more implicit processes. Interventions that are especially designed to modify attentional bias are known as attentional bias modification (ABM) interventions, and one of their advantages is their possible delivery via the computer, making them easily available and easy to add to other face-to-face or computer-based treatments.

**Fig. 1:**
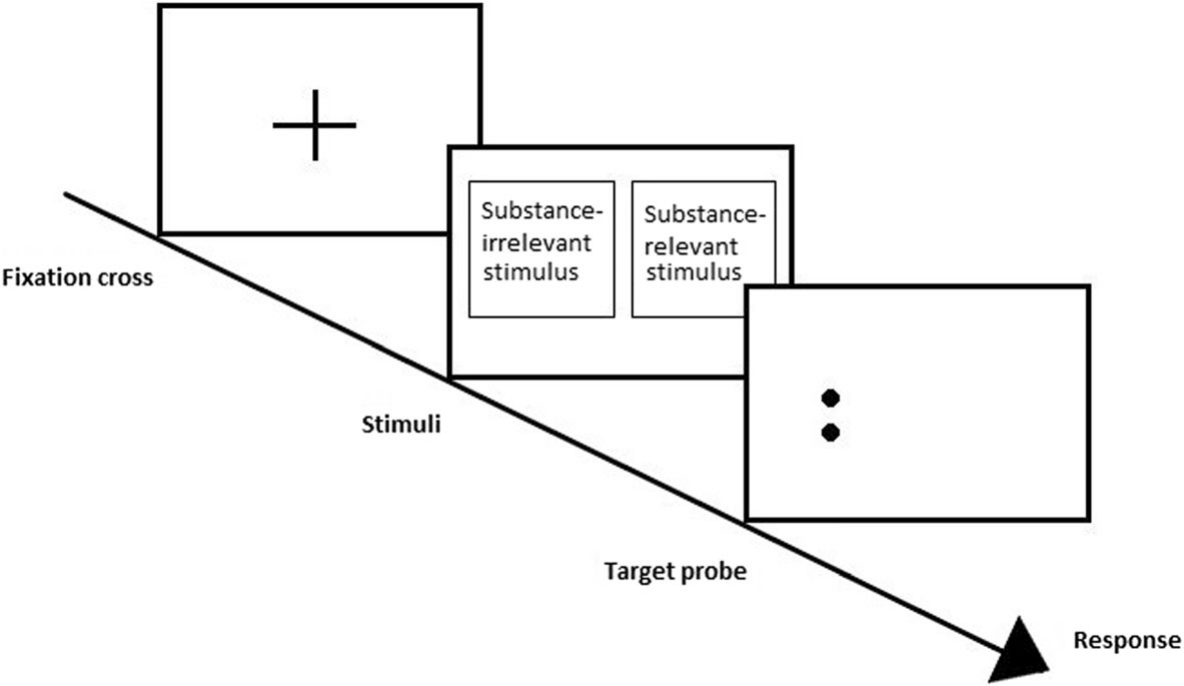
Sample trial of the (modified) dot probe task. After the fixation cross, two stimuli are simultaneously presented on the screen. Thereafter, the target probe appears behind the substance-irrelevant stimulus

**Fig. 2:**
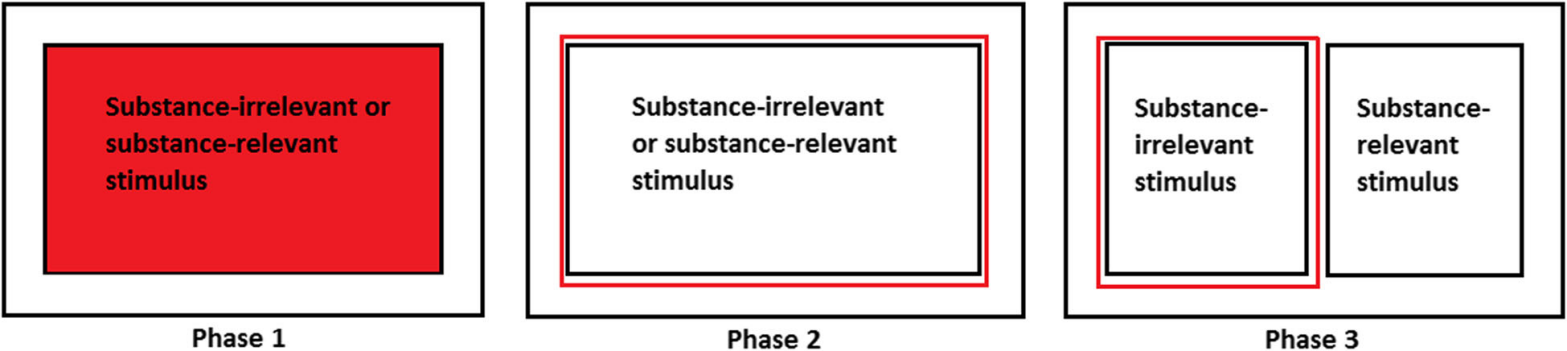
Trial samples of each phase of the Alcohol Attention Control Training Programme. In phase 1, substance-relevant and substance-irrelevant stimuli are successively presented on the screen, while the coloured background of the stimulus needs to be identified. In phase 2, instead of the background, a coloured outline needs to be identified. In the crucial phase, phase 3, substance-relevant and substance-irrelevant stimuli appear simultaneously and the coloured outline of the substance-irrelevant stimulus needs to be identified

Different ABM interventions have been used to modify attentional bias; the most utilised intervention is based on the dot probe paradigm [11, 12], originally meant to measure rather than modify attentional bias. In the adapted task, two pictorial stimuli, one containing substance-relevant information and one containing substance-irrelevant information, are simultaneously presented in the screen. Then, both stimuli disappear and the probe mainly or always (different ratios have been used) appears behind the substance-irrelevant stimulus. Individuals are instructed to identify the position of the probe as quickly as possible with the keyboard or a response box (see Fig. 1). As a result, participants learn to shift their attention towards the substance-irrelevant stimuli and away from the substance-relevant stimuli. Therefore, attentional bias for substance-related cues is meant to be re-trained. Another ABM intervention that has been used in addiction is the Alcohol Attention Control Training Programme (AACTP; [13]). On one hand, this ABM intervention is aiming at reducing speeded detection of substance use-related stimuli, and on the other hand aiming at decreasing the time that people with symptoms of substance use disorder attend to substance-related stimuli once detected. The AACTP consists of three different phases. First, one by one a pictorial stimulus with a coloured background, either substance-relevant or substance-irrelevant, is presented on a computer screen. The content of the pictures needs to be ignored while identifying the colour of the background, using either the keyboard or a response box. Second, again, substance-relevant and substance-irrelevant stimuli are successively presented on the computer screen. In contrast to phase 1 in which pictures have a coloured background, pictures have a coloured outline that needs to be identified. In phase 3, one needs to identify the outline colour of the substance-irrelevant stimulus, while this stimulus is simultaneously presented next to a substance-relevant stimulus (see Fig. 2). This way, participants learn to control their tendency to automatically direct their attention towards the substance-relevant cue.

One of the most important questions when new treatments are developed is whether they are effective in the way they are meant to. In the case of ABM interventions, it is thus important to show that these interventions are successful in (i) reducing attentional bias towards substance-related substances and as a result (ii) leading to clinically relevant symptom reduction. Until today, there are two reviews that addressed the question whether ABM interventions are effective in addiction. First, a recent review evaluated the clinical potentials of ABM interventions in addiction and concluded that the evidence for the effectiveness in reducing substance use-related symptoms is mixed [14]. Furthermore, the authors discussed a couple of methodological issues and statistical limitations of the studies included. Although this review made an important contribution to this field of research, it zoomed in on the potential impact of ABM on the reduction of symptoms and did not incorporate attention to the presence/absence of baseline attentional bias and its changes from baseline to post-test. It might be that the mixed evidence, as shown in the review, can be attributed to the fact that the ABM interventions of several studies did not reduce attentional bias and therefore did not result in a reduction of addition-related symptoms. Several authors emphasised the importance of distinguishing between ABM as a procedure and ABM as a process [15]. That is, the ABM intervention (the procedure) is meant to modify attentional bias (the process). Therefore, changes of symptoms would be expected to go hand in hand with changes of attentional bias. It seems therefore important to not only look at changes of symptoms, but simultaneously also look at changes of attentional bias itself.

Second, a recent meta-analysis examined the efficacy of different types of cognitive bias modification (CBM) interventions in addiction, among which 12 studies examined the effectiveness of ABM interventions [16]. This meta-analysis also focussed on the clinical potentials of these kinds of newly developed interventions and overall found no significant effect of CBM interventions on addiction-related variables. In line, when the authors separately looked at the effectiveness of different types of CBM interventions (i.e. ABM), no significant effects were found. However, on an important note and as mentioned by the authors themselves, the statistical power for these subgroup analyses was rather small. Furthermore, it has been argued that Cristea and colleagues [16] possibly did not find overall significant effects, because no distinction was made between experimental lab studies and clinical trials. As has been argued in comments on their article by Field and colleagues [17] and Wiers [18], it is important to distinguish between lab studies, mainly aiming at the exploration of underlying processes, and clinical trials that are more focussed on clinically relevant changes of symptoms.

To follow up on these publications, this systematic review aims to give an overview of the current status of ABM interventions in addiction and to provide directions for future research. The following questions will be the point of focus: Are current ABM interventions able to successfully modify attentional bias, and are current ABM interventions (also) successful in decreasing symptoms of different substance use disorders? To answer these questions, published peer-reviewed studies were assessed with respect to effects of ABM interventions (i) on changes in attentional bias and (ii) on changes in symptoms, such as substance use, substance dependency, substance use-related problems, craving, and relapse. Furthermore, baseline levels of attentional bias were taken into account when elaborating on the effectiveness of the ABM intervention. In addition, we separately looked at the effectiveness of studies including the general population (lab-studies) and trials including a clinical population.

## Methods

**Fig. 3:**
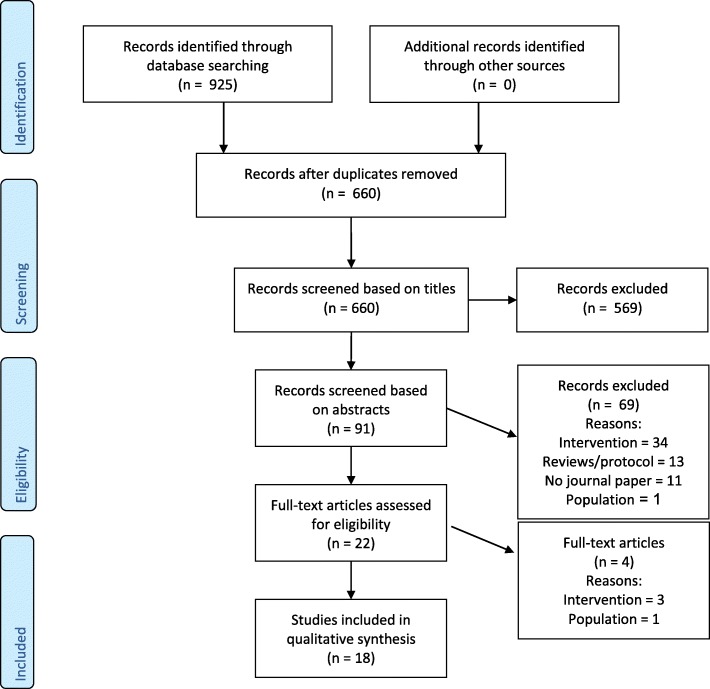
Flowchart of the systematic search

This systematic review was submitted in the PROSPERO register (registration number CRD42016046823), and the review protocol can be assessed here https://www.crd.york.ac.uk/PROSPERO/. Throughout the study, the PRISMA guidelines were followed. See Fig. 3 for the PRISMA flowchart and Additional file 1 for the PRISMA checklist.

### Search strategy and selection criteria

To identify all published peer-reviewed articles, the following databases were systematically searched: PsycINFO, PubMed, and ISI Web of Science. Before conducting the search, search strings were developed based on the two most relevant concepts of this review—attentional bias modification and substance use disorder—by screening several key articles and using Medical Subject Headings (MeSH) terms and related descriptors, as identified by the databases. Each search term was tested individually and also in combination with keywords of the other concept. The search strategy was tested several times and adapted to identify the maximum number of relevant articles. Finally, we used the following key terms for the intervention of interest: attentional bias, attention* bias modification, attention* bias intervention, attention* bias program*, attention* bias therapy, ABM, attention* training, attention* retraining, attention* re-training, cognitive bias modification, cognitive bias intervention, cognitive bias program, cognitive bias training, cognitive bias therapy, CBM, attention* modification, bias modification, and experimental manipulation. Search terms used for substance use disorder were the following: substance use disorder, drug us*, drug abus*, alcohol abus*, drug dependen*, inhalant abuse, polydrug abuse, drug addiction, heroin addiction, drug addiction, substance us*, addiction, substance abus*, alcohol us*, alcohol drinking, alcoholism, tobacco*, nicotine, heavy drink*, and alcohol depend*. A methodology expert assisted in finding the optimal Boolean search strings for each database. Therefore, all related terms were combined using the Boolean logical operators AND and OR. Additionally, reference lists of relevant articles were searched to identify other available peer-reviewed publications. The search was conducted in August 2016 without any restriction for the publication period.

Peer-reviewed articles were included in the current review if they assessed the efficacy of an ABM intervention in order to manipulate attentional bias and to reduce substance use-related symptoms of heavy-using adults (18 years or older) and treatment-seeking individuals with symptoms of substance use disorder. Heavy use was defined either as daily use of substances or as the amount of used substances that was above a (inter-)national cut-off as defined by the studies. Participants who searched for treatment and were treated for a particular addiction were assumed to have symptoms of substance use disorders. As the number of studies in this field of research is limited, all types of study designs and different types of samples (i.e. general population and clinical population) were included. Concerning the design of the study, the only restriction was that studies should have included measurements before and after the intervention, i.e. at least one pre-test and one post-test to help answering our research question. However, to keep the group as homogeneous as possible, studies including participants under the age of 18 and/or diagnosed with any kind of behavioural addiction (e.g. gambling disorder or internet addiction) rather than substance use disorder were excluded from this review.

The main outcome measures were the effectiveness of ABM interventions as measured by changes in attentional bias and symptoms of substance use disorder or heavy use, such as substance use, dependency, substance use-related problems, craving, and time until relapse. No other outcome measures were specified.

### Data extraction and quality assessment

All articles that resulted from the literature search were assessed for eligibility by two independent assessors (JH, MEvHR), who are both content area experts. After removing all duplicates, the eligibility screening took place in three steps based upon the inclusion and exclusion criteria: titles, abstracts, and full-text screening. In steps 2 and 3, the reason for exclusion was noted. After each step, both assessors discussed any disagreement, and if needed, a third person (PJdJ) was asked. Interrater reliability was calculated for each step using Cohen’s kappa.

The following data were extracted from the studies included in this review: (i) general information, such as name of the authors and year of publication; (ii) information about the study design, the follow-up period (if applicable), the duration of the intervention period, the type of ABM intervention, the number of sessions, and the type of control intervention (if applicable); (iii) information about participants, such as sample size, mean age, gender, general or clinical population, and characteristics of the control group (if applicable); (iv) presence/absence of baseline attentional bias; (v) outcome measures from at least one pre-test and one post-test (and possibly follow-up assessments), in particular changes in attentional bias and changes in disorder-related symptoms; and (vi) results of the ABM intervention and key conclusions of the authors. The data from the included studies was extracted by the first review author, and the second author checked the extracted data. Disagreements were resolved by discussion between the two review authors; if no agreement could be reached, it was planned a third author would decide. Furthermore, the quality of the included studies was assessed using Downs and Black’s Study Quality Appraisal Checklist [19]. This checklist was chosen because it is specifically developed to assess the quality of different study designs, in particular to assess randomised as well as non-randomised studies. The original checklist consists of 27 items and has four subscales: reporting, external validity, internal validity, and power of the study. As recommended, a further item was added to the existing checklist to assess baseline comparability [20]. In addition, due to lack of clarity concerning the original item measuring power, this item was restructured to conform with the other items. The question, whether the study has sufficient power to detect a clinically important effect, was answered on a 3-point scale (yes/no/unable to determine). If the influx of participants conformed to the reported power analysis, the question was answered with ‘yes’. ‘No’ was scored if the amount of participants was below the reported power analysis, and if no power analysis was reported, the question was scored as ‘unable to determine’. The assessment was done by two independent assessors (JH, ECB), disagreement was solved by discussion, and if needed, a third person (PJdJ) was asked to give an opinion.

### Data synthesis

Studies were elaborated according to their effects on changes of attentional bias and changes of substance-related symptoms. This was done by taking baseline levels of attentional bias into account. In line with our study protocol, the study findings were structured by the type of addiction and by the type of ABM intervention. In addition to our protocol, we looked at the differences between studies including the general population (lab-studies) and studies including a clinical population as this turned out to be an important distinction [17, 18]. No meta-analysis was planned as a preliminary search indicated that the number of eligible studies within each category would be extremely limited. In line, we think that combining all study results, without taking differences in substance use disorder, type of ABM intervention, and type of population into account, is of little value. Therefore, this review focusses on a narrative synthesis of results, emphasising similarities and differences between studies and suggesting directions for future research.

## Results

After removing the duplicates, the systematic search resulted in a total of 660 papers. The flowchart shows the screening process (see Fig. 3). In the first screening round, 569 articles were excluded, mainly because the papers were not related to either an ABM intervention or any kind of substance use disorder. All papers from which the content was not identifiable were kept in for the next screening round. After the abstracts were screened, 22 articles were left for the full-text screening. The main reasons for excluding papers based on the abstracts were that studies investigated interventions other than ABM or were measuring attentional bias rather than modifying it. During the full-text screening, another four articles were excluded, three because they did not investigate any kind of ABM intervention and one study because the included sample was not a sample of heavy users. By searching the reference lists of relevant papers by hand, no additional papers were found. Finally, 18 papers were included in the current review. Cohen’s kappa for the title screening was *K* = 0.50 (*CI* = 0.39–0.61), *K* = 0.86 (*CI* = 0.74–0.99) for the abstract screening, and *K* = 0.81 (*CI* = 0.47–1.0) for the full-text screening. In other words, the interrater reliability varied from moderate to almost perfect. Most of the included studies are randomised trials, including two [21–32], three [33, 34], four [35], or five [36] groups, whereas two studies have a non-randomised design [13, 37]. Two of the randomised studies used a randomised control trial design [22, 31]. One of the non-randomised studies investigated changes in three groups that were constituted depending on the amount of used alcohol [13], whereas the other included a healthy control group through which randomisation was not possible [37]. There were 13 studies that included participants from the general population (e.g. via a student pool or advertisement in newspapers), and five of the included studies recruited participants from a clinical population. See Table 1 for an overview of the characteristics of the included studies.

**Table 1:** Main characteristics of identified publications

Authors, year	Study	Participants				ABM intervention			Outcomes		Findings
	Design; country	Characteristics	Substance use disorder	*N*	Gender; mean age	Method	Amount of sessions	Duration	AB measure	Symptom measures	
Attwood et al., 2008 [21]	Randomised trial; 2 groups (attend and avoid); UK	Current smokers, smoking at least 5 cigarettes per day	Nicotine	54	56% male; 22	Modified visual probe task (pictures; 500 ms)	1	–	Visual probe task (pictures; 500 ms)	FTND; QSU-Brief	AB at baseline; sig. changes of AB in avoid group; sig. difference in subjective craving between attend and avoid group in males only
Begh et al., 2015 [22]	Double-blind randomised controlled trial; 2 groups (ABM and placebo); UK	Current smokers, smoking at least 10 cigarettes a day	Nicotine	118	–; 45	Modified visual probe task (pictures; 500 ms)	5	5 weeks	Visual probe task (pictures; 500 ms); pictorial Stroop task	MPSS-C; abstinence; time until relapse	No AB at baseline; no sig. changes of AB; no difference between groups in craving; abstinence and time until relapse
Charles et al., 2015 [37]	Non-randomised trial; 4 groups (patient and healthy controls, both assigned to either ABM or placebo); UK	Opiate users in treatment prescribed a substitute medication; healthy controls	Opiate	44	Mainly male; 38–45 (reported per group)	Modified visual probe task (pictures)	1	Unclear	Visual probe task (pictures; 200, 500 ms)	Subjective craving (3 VAS scales)	No AB at baseline; no sig. effect on AB or craving
Cox et al., 2015 [35]	Randomised trial; 4 groups (ABM, motivational intervention, ABM + motivational intervention, control group); UK	Adults drinking above the UK Department of Health cut-off of healthy drinking	Alcohol	148	49% male; 29	Alcohol Attention-Control Training Programme (pictures)	4	4 weeks	Alcohol Stroop task (words)	DRQ; SIP; RTCQ	AB at baseline and changes of AB not reported; alcohol consumption reduced in ABM group
Elfeddali et al., 2016 [23]	Randomised trial with 2 groups (ABM and placebo); Netherlands	Adults smoking on daily basis for at least 1 year	Nicotine	434	31% male; 41	Modified visual probe task (web-based; pictures; 500 ms)	6	Within 2 weeks	Visual probe task (pictures; 500 ms)	FTND; craving; intention to quit smoking	AB at baseline; no sig. changes of AB; effects on abstinence in subsample of heavy smokers
Fadardi and Cox, 2009 [13]	Non-randomised trial with 3 groups (social drinkers, harmful drinkers, hazardous drinkers); UK	Social, hazardous, and harmful adult drinkers	Alcohol	40; 68; 92	14% male, 28% male, 87% male; 30, 23, 41	Alcohol Attention-Control Training Programme (pictures)	0; 2; 4	4 weeks	Alcohol Stroop task (words)	RTCQ; TAAD; SIP	AB at baseline (larger in harmful and hazardous drinkers than in social drinkers); sig. changes of AB in harmful and hazardous drinkers; harmful drinkers showed reduced alcohol consumption and increased readiness to change
Field and Eastwood, 2005 [24]	Randomised trial with 2 groups (attend and avoid); UK	Adult heavy drinkers, drinking at least 14 units (women) or 21 units (men) of alcohol per week on average	Alcohol	40	50% male; 22	Modified dot probe task (pictures; 500 ms)	1	–	Visual probe task (pictures; 500 ms)	AUDIT; DAQ; craving (pre/post training); taste test	No AB at baseline^a^; sig. changes of AB in avoid group; no effects on urge to drink and desire for alcohol; attend group consumed more alcohol than avoid group in taste test
Field et al., 2007 [33]	Randomised trial with 3 groups (attend, avoid, and control); UK	Adults drinking above the UK Department of Health ‘safe’ cut-off of healthy drinking	Alcohol	60	43% male, 43% male, 67% male; 22, 22, 26	Modified dot probe task (pictures; 500 ms)	1	–	Visual probe task (pictures; 500 ms); alcohol Stroop task (words)	Alcohol use disorders identification test; DAQ; urge to drink; taste test	No AB at baseline; sig. changes of AB in avoid group without generalisation to new stimuli and different task; no group difference in alcohol consumption, urge to drink, and consumption of beer in taste test
Kerst and Waters, 2014 [25]	Randomised trial with 2 groups (ABM and control group); US	Current smokers, smoking 10 or more cigarettes per day for the past 2 years	Nicotine	60	50% male; 43	Modified dot probe task (pictures; 500 ms)	21 (3 each day for 7 days)	1 week	Visual probe task (pictures; 500 ms)	QSU; craving; cigarettes smoked per day; physical measures	AB at baseline; sig. changes of AB in ABM group; no effect on smoking behaviour; sig. effect on cued craving but not on non-cued craving in ABM group
Lee and Lee, 2015 [26]	Randomised trial with 2 groups (ABM and psychoeducation); South Korea	Adult problem drinkers as identified with the AUDIT	Alcohol	43	40% male; 22	Modified dot probe task (pictures; 200, 400, 600, 800, 1000 ms)		–	Free-viewing task with eye-tracker (pictures; 1000 ms)	Consumed alcohol during last month; AAAQ; AUDIT; RTCQ	AB at baseline; sig. changes of AB in ABM group; no effect on readiness to change
Lopes et al., 2014 [34]	Randomised trial with 3 groups (3 sessions ABM, 1 session ABM and placebo); Brazil	Adult smokers from a smoking cessation programme, smoking at least 5 cigarettes a day	Nicotine	67	65% male; 45	Modified dot probe task (pictures; 50, 500, and 2000 ms)	1 or 3	2 weeks	Visual probe task (pictures; 50, 500, 2000 ms)	FTND; level of carbon monoxide; Smoking Urge-Brief	AB at baseline; sig. changes of AB in all groups after 24 h; reduction of AB maintained for 6 months after 3 sessions of ABM; no effect on craving and number of smoked cigarettes
Mayer et al., 2016 [27]	Randomised trial with 2 groups (ABM and placebo); US	Treatment-seeking adults diagnosed with cocaine use disorder used in at least 4 of prior 30 days	Opiate	40	63% male; 38	Modified dot probe task (pictures; 200, 500 ms)	5	4 weeks	Visual probe task (pictures; 200, 500 ms)	Cocaine use; CCQ-G; CSSA; FTND; BIS; AUDIT; BDI-2; STAI-T	No AB at baseline; no sig. changes of AB; no effect on craving, or drug use behaviour
McGeary et al., 2014 [28]	Randomised trial with 2 groups (ABM and placebo); US	Heavy drinking students as identified with the AUDIT	Alcohol	41	100% male; 19	Modified dot probe task with words (500 ms)	8	4 weeks	Not assessed	DHQ	No AB at baseline reported an no changes in AB assessed; reduced amount of alcohol consumption in ABM group
McHugh et al., 2010 [29]	Randomised trial with 2 groups (ABM and placebo); US	Adult smokers, smoking at least 10 cigarettes a day	Nicotine	64	65% male; 38	Modified dot probe task (pictures; 500 ms)	1	–	Visual probe task (pictures; 500 ms)	FTND; TLFB; QSU-Brief	No AB at baseline; no sig. changes of AB; no effect on craving
Schoenmakers et al., 2007 [30]	Randomised trial with 2 groups (ABM and placebo); Netherlands	Heavy drinking students as identified with a self-report questionnaire	Alcohol	106	100% male; 21	Modified dot probe task (pictures; 500 ms)	1	–	Visual probe task (pictures; 500 ms); flicker task	Preference test; craving	No AB at baseline^a^; smaller AB in ABM group compared with control group at post-test without generalisation; no effect on craving and preference test
Schoenmakers et al., 2010 [31]	Randomised trial with 2 groups (ABM and placebo); Netherlands	Adults diagnosed with alcohol dependence	Alcohol	43	77% male; 45	Modified dot probe task (pictures; 200, 500 ms)	5	3 weeks	Visual probe task (pictures; 200, 500 ms)	DAQ; Time to relapse	No AB at baseline^a^; sig. changes of AB in ABM group; no effect on craving, but time until relapse longer in ABM group
Wiers et al., 2015 [36]	Randomised trial with 5 groups (4 experimental conditions and one placebo group); Netherlands	Heavy drinking adults as identified with the AUDIT	Alcohol	314	-; 48	Alcohol Attention Control Training Programme	4	2–14 days	–	Alcohol consumption; craving; RCQ	No AB at baseline and changes in AB assessed; reduction of drinking and craving, but this was found in all conditions including control group
Ziaee et al., 2016 [32]	Randomised trial with 2 groups (ABM + TAU and TAU only); Iran	Adults undergoing methadone maintenance therapy	Opiate	48	100%; 33 in experimental group, 39 in control group	Drug attention control training (words and pictures)	3	2 weeks	Drug-Stroop task (words)	SCQ; RTCQ; PSS; Persian drug temptation questionnaire	No AB at baseline reported; sig. changes of AB in ABM group; decreased doses of medicine and number of lapses and increase in readiness to change in ABM group

^a^Based on calculations from data derived from tables or figures (see supporting information)

### Type of ABM intervention

The dot probe task has most often been used to modify attentional bias. In particular, 14 of the studies used a modified version in order to change attentional bias. The used versions slightly differed in some aspects. First, the ratio of the dot replacing either the substance-related stimulus or the neutral stimulus differed between studies. In most of the versions, the dot always appeared behind the neutral stimulus, whereas, for example one study used a 80:20 ratio [26]. Second, whereas most studies used a pictorial dot probe task, one study used words instead of pictures, which were personalised in the sense that participants could choose the words that were most relevant to them [28]. Three of the studies used the Alcohol Attention Control Training Programme to modify attentional bias in alcohol use disorder [13, 35, 36]. One study in opiate use disorder used an adapted version of this training, tailored for participants using opiates [32].

There was a great variety in the number of provided ABM sessions. Whereas seven studies investigated the effects of a single session, the other studies tested the effects of a multi-session ABM intervention. The number of sessions varied from three to eight, and the time interval in which the sessions were provided varied from 1 to 5 weeks.

### Substance use disorders

There were three different substance use disorders in which ABM interventions have been investigated. The majority of papers investigated the effects of ABM intervention in alcohol use disorder or people using alcohol heavily (*n* = 9). Nicotine dependence was studied in six of the included papers. Lastly, three of the studies investigated the effects of ABM intervention in opiate use disorder.

### Outcome measures

Changes in attentional bias were investigated in 16 of the 18 studies. Most of the studies measured attentional bias with the dot probe task (*n* = 9). Another three studies used the dot probe task in combination with either an adapted version of the Stroop task (*n* = 1), the flicker task (*n* = 1), or the Stroop task and the flicker task (*n* = 1). Two studies investigated changes in attentional bias with the alcohol Stroop task and one study used a modified version, called the drug Stroop task. Lastly, one study used a free-viewing task with an eye tracker. A variety of outcome measures has been used to investigate changes in substance use-related symptoms. Most of the studies used self-report measures in the form of questionnaires. Most frequently used measures were craving (*n* = 10) and the amount of used substance either directly after the intervention or within a certain period of time (*n* = 8). Other measures were time until relapse (*n* = 2) or number of relapses (*n* = 1), readiness to change (*n* = 2), and abstinence (*n* = 2). The majority of studies investigated more than one outcome measure to indicate changes of substance use-related symptoms.

### Study findings

See Table 1 for an overview of the characteristics of the included studies and the main study findings.

#### Alcohol

There were six studies testing the effects of a modified dot probe task in heavy drinking or alcohol use disorder, and three studies investigated the effects of the AACTP.

##### Dot probe task training

Three of the six studies found a decline in attentional bias but no effect on alcohol use-related symptoms. First, Field and colleagues [33] tested the effects of a single-session ABM intervention by comparing three groups: one group received a modified dot probe task (100%; avoid group), one group in which the alcohol-related pictures were always replaced by the probe (attend group), and one control group (standard visual dot probe task). At baseline, in none of the groups a significant attentional bias for alcohol-related stimuli was found. In the avoid group, attentional bias declined from baseline to post-test, whereas in the attend group, attentional bias increased and in the control group, no significant change was found. The decline of attentional bias in the avoid group did not generalise to new stimuli, but unexpectedly, an increase of attentional bias from baseline to post-test for new stimuli was measured. There was no generalisation of changes in attentional bias. Furthermore, there was no effect of ABM intervention on subjective craving and the amount of consumed beer in a post-taste test. Second, Lee and Lee [26] found similar results, by comparing a single session of ABM intervention (80:20 ratio) with a group of participants who received psychoeducation in the form of a booklet. At baseline, attentional bias was present at 200–400 ms, 400–600 ms, and 800–1000 ms in both groups (see Additional file 2). At 600–800 ms, the control group showed a significant attentional bias, whereas attentional bias in the ABM group approached significance. There were no significant differences between groups at baseline. In the ABM group, attentional bias significantly declined from baseline to post-test. This effect was attributed to changes in the 200–400-ms, 400–600-ms, and 800–1000-ms condition of the ABM intervention from pre-test to post-test. In the psychoeducation group, there was no decline of attentional bias. In the ABM group, there was no alteration in readiness to change as measured with the Readiness to Change Questionnaire, while an increase in readiness to change was found in the psychoeducation group. Lastly, Schoenmakers and colleagues [30] compared a single session (96:4 ratio) with a control group that did a standard dot probe task. At baseline, attentional bias scores of the ABM group were not significantly different from the control group, and based on the descriptives our calculation indicated that there was no attentional bias for alcohol-related cues in both groups (see Additional file 2). At the post-test, the ABM group had significantly lower attentional bias scores than the control group. Furthermore, there was no reduction of attentional bias to novel stimuli. No changes in subjective craving (urge to drink) in the ABM group or differences between the groups on a preference taste test were found.

Another two studies found positive effects of multiple sessions of ABM intervention on alcohol-related symptoms. However, due to insufficient information on baseline attentional bias and/or changes of attentional bias from baseline to post-test/s, it is unclear whether these effects can be attributed to a change in attentional bias. First, McGeary and colleagues [28] tested the efficacy of eight personalised ABM sessions (100%), compared with the standard visual dot probe task. Unfortunately, there was no assessment of attentional bias for alcohol-related stimuli, and therefore, no changes from baseline to post-test could be reported. The ABM group showed a reduction of the amount of consumed alcohol at post-test while there was no difference in consumption in the control group. Second, Schoenmakers and colleagues [31] tested the effects of five sessions (100%) in a clinical sample. This group was compared with an active control group that received the standard visual dot probe task. No differences between groups in attentional bias were found at baseline, but it was not reported whether an attentional bias for alcohol-related cues was present, as means of baseline attentional bias were only presented graphically. Our estimation revealed that there was no attentional bias in both groups (see Additional file 2). The ABM group showed a decline in attentional bias scores in the 500-ms condition from baseline to post-test while there was no change in the control group. Note that changes were only assessed within each group, and as a result, it is not clear whether changes over time were different between groups. Although there were no differences in subjective craving for alcohol between the ABM group and the controls at post-test, the time until the first relapse was significantly longer in the ABM group (note that this analysis was based on only eight patients).

Lastly, there was one study in which ABM intervention led to changes in attentional bias and alcohol-related symptoms. Field and Eastwood [24] compared the effects of one session (100%; avoid group) with a group that received one session in which all probes appeared behind the alcohol-related stimulus (attend group). At baseline, no difference between groups in attentional bias was found, but it was not reported whether attentional bias for alcohol-related stimuli was present. Based on the graphical presentation of the means and standard errors, we estimated that attentional bias was absent (see Additional file 2). In the avoid group, attentional bias scores significantly declined from baseline to post-test and differed significantly from the attend group that showed a significant increase in attentional bias scores from baseline to post-test. There was no difference from baseline to post-test between groups on urge to drink, or desire for alcohol, but a significant difference in the amount of consumed alcohol was found. That is, the attend group consumed more beer than the avoid group in the taste test. However, given the absence of a control group (e.g. who received a standard dot probe task), the results need to be interpreted with caution.

##### AACTP

All three studies that tested the effects of the AACTP in alcohol found positive effects on alcohol-related symptoms. However, it was unclear whether these effects can be attributed to a change in attentional bias, as there was insufficient information on baseline attentional bias and/or changes of attentional bias. First, Cox and colleagues [35] compared four sessions of ABM intervention with a short motivational intervention, called Life Enhancement and Advancement Programme (LEAP). One group received four sessions of AACTP, the second group received four sessions of LEAP, the third group received AACTP and LEAP, and the last group was a control group that received no intervention. Results of the baseline assessment as well as changes from baseline to post-test/s were not reported. At the post-test, the ABM group showed a marginally significant reduction of the amount of weekly used alcohol during a regular week, but not yet a reduction in the mean quantity of consumed alcohol during an atypical week. However, the mean quantity of consumed alcohol during an atypical week in the ABM group declined from post-test to 3-month and 6-month follow-ups. The reduction of the amount of weekly used alcohol during a regular week lasted until the 3-month follow-up assessment. Another 3 months later at the 6-month follow-up, this effect disappeared. In the LEAP group, there was no decline of the amount of weekly used alcohol from baseline to post-test, but at the 3-month and 6-month follow-ups, a significant decline for used alcohol during regular and atypical weeks was found. There were no additional benefits of combining both interventions. Second, Fadardi and Cox [13] compared the effects of the AACTP among three groups: social drinkers, harmful drinkers, and hazardous drinkers. The last group received four sessions of AACTP, whereas the harmful drinkers received two sessions of the same training. The social drinkers received no training. There was no active control group included in this study. Attentional bias at baseline was found to be larger in hazardous and harmful drinkers than in social drinkers. Both, hazardous and harmful drinkers showed a reduction in attentional bias scores from baseline to post-test. However, it was not clear whether this change can be ascribed to the intervention, as no adequate control condition was present for each group. In addition, all groups were assessed separately, meaning that it is unclear whether changes over time were different/same between groups. The effect in the group of the hazardous and harmful drinkers did not last until the 3-month follow-up assessment. In the group of hazardous drinkers, an increase in readiness to change was found. Furthermore, a reduction in alcohol consumption was found in the group of harmful drinkers which lasted until the 3-month follow-up. The third study compared four sessions of web-based AACTP with three different versions of a web-based approach bias modification training and one control group that did a placebo intervention based on the paradigm of the approach bias modification training [36]. Attentional bias for alcohol-related cues and its changes from baseline to post-test/s was not assessed. In all five groups, a reduction of alcohol consumption, craving, and self-efficacy was found. Whereas AACTP lead to the smallest effects on alcohol consumption—the reduction was only significant from baseline to post-test but did not last until the first follow-up—the effects on self-efficacy were strongest when compared to the other groups.

#### Nicotine

All studies in nicotine dependency were done with a modified version of the dot probe task, and three of the six studies found no effect on either changes in attentional bias or changes in nicotine use-related symptoms. First, Begh and colleagues [22] investigated the effects of five sessions (92:8 ratio) via the internet in a clinical sample, compared with a group that received a standard visual dot probe task At baseline, there was no attentional bias for nicotine-related stimuli in both groups. Individuals in the training group showed no changes in attentional bias from baseline to all post-measurements (4, 8, 12, and 24 weeks). There were no changes in symptom-related measures including craving, abstinence, and time until relapse. Second, Lopes and colleagues (2014) compared the effects of an ABM intervention (100%), with the effects of a placebo intervention, i.e. standard visual probe task. They randomly allocated participants with a nicotine dependence to different conditions (group 1: three sessions of ABM intervention; group 2: two sessions of ABM intervention and one session of placebo intervention; group 3: three sessions of placebo intervention). At baseline, all groups showed an attentional bias for nicotine-related stimuli, as indicated by a significant *t* test against zero. They found that attentional bias for nicotine-related stimuli was significantly lower and became negative 24 h after intervention. However, this change could not be ascribed to the effects of the ABM intervention as these changes did not differ between groups. Nevertheless, it seems that the amount of provided ABM sessions had an influence of the duration of the changes. That is, changes of attentional bias lasted longest in the group who received three sessions of ABM intervention. There was no effect of the ABM intervention on the number of smoked cigarettes per day and subjective craving. Third, McHugh and colleagues [29] also compared the effects of a modified pictorial visual dot probe task (85:15 ratio) with a placebo training (standard pictorial visual dot probe task). In line with Begh and colleagues [22], they found no significant attentional bias for nicotine-related stimuli at baseline and no changes of attentional bias after one session of ABM intervention when comparing baseline scores with post-test scores. Furthermore, there was no difference between groups in subjective craving at post-test.

Another study, by Elfeddali and colleagues [23], compared one group that received six sessions of ABM intervention (92:8 ratio), with a group that received six sessions of a standard visual dot probe task. All sessions as well as the assessments were delivered via the internet. At baseline, there was an attentional bias for nicotine-related stimuli in all groups. In the first instance, no effects of the training on attentional bias or substance use-related symptoms were found. However, post hoc analyses in a subsample of heavy smokers revealed a positive effect of the training on abstinence when compared with light and moderate smokers. Yet, changes in attentional bias remained non-significant.

Another two studies found effects of ABM intervention on changes in attentional bias and nicotine use-related symptoms. First, Attwood and colleagues [21] compared one session (100%) with a group that received the same intervention with the exception that all probes appeared behind the nicotine-related pictures (attend group). At baseline, both groups showed a significant attentional bias for nicotine-related cues. At post-test, both groups differed significantly from each other. There was a significant decline in attentional bias from baseline to post-test in the avoid group that was not observed in the attend group. Furthermore, in male participants, there was a marginally significant difference between groups with regard to subjective craving. That is, both groups showed an increase of craving to smoking stimuli at post-exposure, but this increase was smaller in the avoid group than in the attend group. Female participants did not differ in subjective craving as both groups showed an increase from baseline to post-exposure. Lastly, Kerst and Waters [25] tested the effectiveness of 21 short sessions of ABM intervention (100%), delivered via a personal digital assistant. This group was compared with a non-intervention control group. At baseline, they found a significant attentional bias for nicotine-related cues. There was a significant decline of attentional bias from baseline to post-test in the experimental group whereas no changes were found in the control group. When craving was induced by a smoking-related stimulus, the ABM group showed significant lower craving ratings at post-test compared to the control group. However, there was no difference between groups in the reduction of non-cued craving and in the amount of smoked cigarettes a day.

#### Opiate

There were two studies investigating the effects of ABM interventions in opiate use disorder with a modified version of the dot probe task, and one study tested the effects of an adapted version of the AACTP.

##### Dot probe task training

Both studies that used a modified dot probe task found effects neither on attentional bias nor on opiate use-related symptoms. First, Charles and colleagues [37] tested the effects of one session (100%) in a four-group design. Participants were diagnosed with opiate dependency (clinical sample) or were healthy controls. Half of both groups were assigned either to the ABM group or to the placebo group (standard visual dot probe task). At baseline, there was no difference in attentional bias for opiate-related stimuli between users and healthy controls. Furthermore, comparing the scores of all four groups from baseline to post-test and from baseline to 1-month follow-up, no changes in attentional bias were found. There was also no effect of ABM on subjective craving on both post-measurements. Second, another study by Mayer and colleagues [27] compared five sessions (100%) with a placebo control group (standard visual dot probe task) in a clinical sample. Similar to the study by Charles and colleagues (2015), they did not find any attentional bias for opiate use-related symptoms at baseline. Furthermore, the ABM intervention had no effect on either changes of attentional bias or the amount of used cocaine, craving, and withdrawal symptoms.

##### AACTP

Ziaee and colleagues [32] compared one group that received ABM intervention and treatment as usual (TAU) with one group that received TAU only. Both groups were drug abusers in treatment for methadone maintenance. The ABM intervention was based on the AACTP but was adapted with stimuli relevant for opiate users, called the Drug Attention Control Training Program. The intervention included pictorial stimuli as well as words. The baseline scores of attentional bias were not reported. Therefore, it is not clear whether attentional bias was present prior to the intervention. Despite this limitation, the authors reported a significant decline in attentional bias from baseline to post-test in the ABM group, which was significantly different from the control group. Furthermore, in comparison with the control group, the ABM group showed an increase in readiness to change as well as a reduction in doses of methadone and the number of relapses. See Table 2 for an overview of all study findings concerning changes of attentional bias and changes of symptoms.

**Table 2:** Study results structured by effects on attentional bias and symptoms

Results	Publications ordered by substance		Amount of sessions	AB at baseline
AB + Symp −	Alcohol:			
		Field et al. 2007 [33]	1	No AB
		Lee and Lee 2015 [26]	1	AB found^a^
		Schoenmakers et al. 2007 [30]	1	No AB^a^
AB − Symp +	Nicotine:			
		Elfeddali et al. 2016^b^ [23]	6	AB found
AB unknown Symp +	Alcohol:			
		Cox et al. 2015 [35]	4	Not reported
		Fadardi and Cox 2009 [13]	4	AB found
		McGeary et al. 2014 [28]	8	Not reported
		Wiers et al. 2015 [36]	4	Not reported
AB + Symp +	Alcohol:			
		Field and Eastwood 2005 [24]	1	No AB^a^
		*Schoenmakers et al. 2010* [31]	5	No AB^a^
	Nicotine:			
		Attwood et al. 2008^c^ [21]	1	AB found
		Kerst and Waters 2014 [25]	21	AB found
	Opiate:			
		*Ziaee et al. 2016* [32]	3	Not reported
AB − Symp −	Nicotine:			
		*Begh et al. 2015* [22]	5	No AB
		Lopes et al. 2014 [34]	1–3	AB found
		McHugh et al. 2010 [29]	1	No AB
	Opiate:			
		*Charles et al. 2015* [37]	1	No AB
		*Mayer et al. 2016* [27]	5	No AB

Studies in clinical population are presented in italics

*AB +* attentional bias significantly changed from baseline to post-test/s, *AB −* attentional bias did not change from baseline to post-test/s, *AB unknown* changes in attentional bias were not reported or unclear, *Symp +* significant change on one or more addiction outcome measures from baseline to post-test/s, *Symp −* addiction outcome measures did not change from baseline to post-test/s

^a^Based on calculations from data derived from tables or figures (see supporting information)

^b^Significant changes in symptoms (abstinence) was only found in subsample (heavy smokers)

^c^Significant changes in symptoms (subjective craving) was only found in subsample (males)

### Study quality

Table 3 presents an overview of the quality assessment as measured with an adapted version of the criteria of Downs and Black [19]. The reviewed studies were of variable methodological quality with total scores ranging from 12 to 23 (maximum of 29) with a mean score of 17.9. Originally, there was no cut-off score to identify low-quality and high-quality papers; however, other researchers introduced a cut-off score of 14 points [38, 39]. Given this cut-off score, most of the papers ranged from low-average to high-average quality, whereas one paper scored below 14 points and was identified as a low-quality paper [28], especially because information was insufficient (e.g. low scores on subscale internal validity due to lack of reporting detailed information).

**Table 3:** Overview of the quality assessment

Authors, year	Reporting (0–11)	External validity (0–3)	Internal validity—bias (0–7)	Internal validity—confounding (0–7)	Power (0–1)	Total (0–29)	Adjusted total^a^ (0–26)
Attwood et al., 2008 [21]	8	0	5	4	0	17 (58.6%)	14 (53.9%)
Begh et al., 2015 [22]	6	1	7	4	1	19 (65.5%)	–
Charles et al., 2015 [37]	10	0	4	1	0	15 (51.7%)	12 (46.2%)
Cox et al., 2015 [35]	9	1	5	3	0	18 (62.1%)	–
Elfeddali et al., 2016 [23]	9	1	6	4	1	21 (72.4%)	–
Fadardi and Cox, 2009 [13]	9	0	5	3	0	17 (58.6%)	–
Field and Eastwood, 2005 [24]	8	1	5	3	0	17 (58.65)	14 (53.9%)
Field et al., 2007 [33]	8	1	7	2	0	18 (62.1%)	15 (57.7%)
Kerst and Waters, 2014 [25]	11	0	6	4	0	21 (72.4%)	18 (69.2%)
Lee and Lee, 2015 [26]	10	1	7	5	0	23 (79.3%)	20 (76.9%)
Lopes et al., 2014 [34]	10	2	5	3	0	20 (69.0%)	–
Mayer et al., 2016 [27]	7	0	5	4	0	16 (55.2%)	–
McGeary et al., 2014 [28]	7	1	3	1	0	12 (41.45)	9 (34.6%)
McHugh et al., 2010 [29]	7	0	4	4	0	15 (51.7%)	12 (46.2%)
Schoenmakers et al., 2007 [30]	10	0	5	4	0	19 (65.5%)	16 (61.5%)
Schoenmakers et al., 2010 [31]	8	1	7	6	0	22 (75.9%)	–
Wiers et al., 2015 [36]	9	1	3	3	0	16 (55.2%)	–
Ziaee et al., 2016 [32]	8	0	5	3	1	17 (58.6%)	–

^a^Studies without follow-up assessment were in first instance scored in favour of their quality, i.e. they received a ‘1’ score on the three follow-up measurements questions. This column represents the adjusted scores after the three follow-up measurement questions were excluded

There were a couple of methodological concerns that were repeatedly identified. First, only three of the 18 studies reported clear and sufficient power analysis [22, 23, 32]. Most of the other studies omitted to calculate or report on power (*n* = 15). Second, although most of the studies included an active control group, only the minority sufficiently reported whether participants and assessors were blinded for the condition of the participants. The other studies missed to report on blinding of participants and assessors (*n* = 11; *n* = 12, respectively). This was reflected in relatively low ratings on the subscale ‘internal validity—confounding’. Third, the source and the representativeness of the sample was often not clearly reported. Therefore, several studies have low ratings on the subscale ‘external validity’.

## Discussion

This systematic review was designed to examine whether ABM interventions are able to successfully modify attentional bias and whether such modification would be associated with a decrease in addictive symptoms. Thus, different from related reviews that primarily focussed on the overall impact of ABM on clinical outcomes [14, 16], the current review addressed in more detail critical aspects of the designs that were used including the assessment of baseline attentional bias and its changes from pre- to post-test and looked at possible differences in outcomes between studies in the general population and clinical studies. Together, this information may facilitate a more nuanced elaboration of the current evidence regarding the effectiveness of ABM interventions and may provide some specific directions for future research. The number of available ABM studies within the realm of substance addiction is still very limited. In addition, the approaches in terms of ABM procedures and AB assessments are highly variable. Furthermore, the groups that are targeted are highly variable both with regard to the type of substance and their clinical status (see also [18]). This variability of study population together with the limited amount of studies investigating ABM interventions impede the possibility of merging and comparing the results in a quantitative manner. Therefore, we decided to restrict this systematic review to a more qualitative analysis to give a more specified and detailed view of the current evidence.

The current systematic review identified 18 studies investigating the effects of ABM interventions in heavy use or substance use disorders. Several studies provided evidence indicating that ABM interventions are able to successfully modify attentional bias and that ABM interventions might have clinically relevant effects on symptoms of addiction, suggesting that ABM might be a valuable addition to current treatments. However, overall, the results appeared to be quite mixed and effects on symptoms of addiction did not systematically go hand in hand with changes of attentional bias. Consistent with this mixed pattern, an earlier subgroup analysis of 12 ABM studies that were part of a larger meta-analysis covering various forms of cognitive bias modification in substance addictions failed to find a meaningful effect of ABM on addiction, whereas at post-test, attentional bias was generally lower (moderate effect size) in the ABM than in the control conditions ([16]; see Additional file 3 for similarities/differences of included studies within the current systematic review and the meta-analysis by Cristea et al. [16]). In addition to this earlier meta-analysis, the findings of the current systematic review further showed that attentional bias was not consistently present at baseline when changes in attentional bias or symptoms of addiction were observed. Furthermore, no clear differences in the effectiveness of ABM interventions were found between studies within the general population and studies in the clinical population. However, given the limited number of clinical studies within this field of research, drawing firm conclusions might be too early.

### Effects of ABM intervention on attentional bias

With the exception of two studies [23, 34]—which did not find unique changes of attentional bias from baseline to post-test in the ABM group—almost all other studies that reported and found attentional bias at baseline also found that ABM intervention resulted in significant changes of attentional bias from baseline to post-test [21, 25, 26]. For one study that found attentional bias at baseline, it was unclear whether it changed [13]. Generally, these results seem to indicate that ABM interventions are able to successfully modify attentional bias if attentional bias for substance-related cues is present prior to the intervention. In accordance, when attentional bias for substance-related cues was not present at baseline, several studies found no modification of attentional bias [22, 27, 29, 37]. However, another four studies found that modifying attention in the desirable direction was also possible when no significant attentional bias for substance-related cues at baseline was found [24, 30, 31, 33]. This finding raises the question whether baseline attentional bias is a prerequisite for the effectiveness of ABM interventions. It might indicate the possibility to train a new bias away from substance-related cues when no bias is present rather than a reduction of a pre-existing bias towards substance-related cues. This new learned tendency to avoid substance use-related stimuli might have a protective quality, for example when it comes to relapse. Possibly, ABM interventions might be able to positively influence symptoms of addictive behaviour via different pathways. It seems important that future research clarifies which mechanisms underlie the effectiveness of ABM interventions. In particular, it appears relevant to investigate whether the reduction of pre-existing attentional bias or the teaching of a new bias is essential for a reduction in symptoms of addiction.

In line, even though the study results in general indicate that the modification of attentional bias using ABM interventions is possible, it is noteworthy that only one third of the included studies found and reported a significant attentional bias for substance-related cues at baseline. The other studies either found no attentional bias (*n* = 8) or due to incomplete reporting it was unclear whether attentional bias was present before the intervention took place (*n* = 4). There were no clear indications that these inconsistencies of baseline attentional bias were related to either type of addiction (alcohol, nicotine, or opiate), context (lab versus clinic versus home environment), or type of participants (general or clinical population). The most intuitive explanation might be that attentional bias in the field of addiction has been overvalued and plays a less profound role than expected. However, given the scope of research that repeatedly found attentional bias for substance-related cues [8, 40], there might be other possible explanations for these ambiguous findings.

One factor that might explain this inconsistency is the non-optimal operationalisation of attentional bias. When examining the way attentional bias was assessed, it stands out that 8 out of 12 studies using the visual dot probe task did not find attentional bias. Perhaps, the visual dot probe task is not sufficiently reliable or not an adequate index of attentional bias. One explanation might be that this task is not optimally suited to differentiate between two important components of attentional bias—engagement and disengagement of attention [41]. Other assessment tasks that are in a better position to disentangle these components of attention are preferable, the more so, because the presence and the strength of attentional bias towards a substance-related cue might be dependent on the momentary evaluation of this cue [40]. In particular, people who want to change their drug use behaviour might develop an approach-avoidance pattern towards the pertinent substance, meaning that initial attention is directed towards the substance, but due to their motivational state after this initial approach, attention is directly directed away from the cue [42]. The avoidance of the cue might mask the initial orientation towards the cue, and therefore, the reaction times that derive from assessments with for example the visual dot probe task might be less clear. To further disentangle the way attention in addiction is directed, the use of other assessment tasks or the combination with an eye tracker might be advisable. In line, we want to point out that finding reliable assessment tasks to measure attentional bias should be one important focus of future research. Second, it might be that the degree of attentional bias varies over time as well as the motivational saliency of substance use might vary over time and contexts. In line with this, a review by Cox and colleagues [43] suggested that attentional bias for substance use-related stimuli is strongest when substance use is of current concern, for example triggered via external cues like posters. Therefore, the context in which attentional bias is assessed and whether substance use is salient might indirectly influence whether attentional bias will be found.

Concerning changes of attentional bias from baseline to post-tests, it is notable that the assessment task and the ABM intervention were often based on the same paradigm. Therefore, it cannot be ruled out that the reported changes merely reflect a restricted learning effect—becoming better on this particular task—rather than a decrease of attentional bias for substance-related cues. Two of the included studies support this idea, showing that the change of attentional bias was only found with the task that equalled the intervention, but not with another assessment task [30, 33]. Future research should therefore consider different paradigms for the assessment task and the intervention to differentiate between direct learning effects and transfer effects that represent the generalisation of newly learned processes. In addition, it has to be taken into account that it appears that multiple sessions might be necessary to achieve long lasting effects of the modification, although even a single session of ABM intervention was found to lead to changes in attentional bias [30, 33, 42]. The study by Lopes and colleagues [34] found that the effect of one session of ABM intervention on attentional bias lasted shorter than the effects of three sessions of ABM intervention. This might imply that the amount of provided sessions contributes to a longer duration of effects, and therefore, multiple sessions are probably needed to modify attentional bias in the long term.

### Effects of ABM intervention on symptoms of substance use disorders

Based on the current results, no clear conclusions can be drawn about whether ABM interventions are effective in reducing symptoms of addiction. Ten out of the 18 included studies reported significant changes of substance use-related symptoms [13, 21, 23–25, 28, 31, 32, 35, 36]. The majority of these studies found these positive effects after having provided multiple sessions, suggesting that clinically meaningful effects of ABM interventions are more likely to occur after a multi-session ABM intervention. Only one of these multi-session ABM studies that found significant changes of symptoms of addiction reported the presence of baseline attentional bias and its successful modification from baseline to post-test [25]. On an important note, based on our elaboration above, this inconsistency in findings might also be due to a poor psychometric quality of current attentional bias measures (e.g. in terms of test-re-test reliability).

Based on the current results, no firm conclusions can be drawn about the specific effects of ABM interventions on symptoms, because a number of different parameters of addiction were used, including abstinence [23], craving [25, 32], amount of consumed alcohol [13], time until relapse [31], and number of lapses [32]. Future research should further investigate which parameters of addiction might be positively influenced by ABM interventions, and therefore, future research might consider including a consistent range of pre-defined outcome measures.

Furthermore, the long-term effects of ABM intervention on changes of symptoms are yet unclear. Only four of the studies that reported positive effects on symptoms after multiple sessions of ABM intervention included follow-up assessments [13, 23, 31, 32]. The follow-up duration varied from 2 to 12 months, and all but one study included only one follow-up assessment. It is not clear how long the positive effects last, but the study by Ziaee and colleagues [32] suggests that the duration might be limited. In line, it is also possible that the effects of ABM intervention as a stand-alone treatment are limited. That is, changing the attentional pattern towards substance-related cues might be essential, but not sufficient to change addictive behaviour permanently. This would suggest that combining ABM interventions with other treatments, for example CBT, might lead to more permanent effects. To clarify which factors influence the lasting of effects of the ABM interventions, future research should consider the inclusion of more than one follow-up assessment and the combination with other interventions. We identified that one factor that might influence the lasting of effects is the amount of training sessions.

### Type of sample: general and clinical population

Looking at the results of studies including the general population (*n* = 13) and studies including a clinical population (*n* = 5), it stands out that no particular differences can be found regarding the effectiveness of ABM interventions. Whereas two of the studies including a clinical population found both, significant changes of attentional bias from baseline to post-test and an effect on substance use-related symptoms, the other three clinical studies found no effect on attentional bias and no changes in symptom measures. It seems also not clear whether the mixed results might be dependent on the type of substance use disorder. There was only one study investigating the effects in alcohol (positive findings) and one study in nicotine (negative findings). The other three studies tested ABM interventions in opiate dependency—with two of these studies without any effect. Given this limited scope of studies in the clinical population, it seems too early to draw any conclusions. More studies are necessary to further explore the clinical relevance of ABM interventions.

### Methodological differences and limitations of included studies

It stands out that baseline measures of attentional bias (*n* = 8) and even changes of attentional bias from baseline to post-test (*n* = 4) were not consistently reported. As argued by Clarke and colleagues [44], the successful modification of attentional bias is a presumption of clinical meaningful changes that can be ascribed to the effectiveness of the ABM intervention. If baseline attentional bias and its modification are not assessed or not reported, the interpretation of results is limited. Measuring attentional bias and its changes from baseline to post-tests therefore serves as a manipulation check of the effectiveness of the ABM intervention, and future research should make sure to report complete results in order to allow drawing firmer conclusions about the direct effects of ABM interventions on addiction.

Another notable aspect is the diversity of the designs and procedures of the studies. First, the studies differed from each other with regard to the context in which ABM interventions were delivered. As was shown in the field of anxiety disorders, the context might impact the effectiveness of the intervention profoundly [45–47]. Given that the effectiveness of ABM interventions in the context of substance use might be dependent on the level of experienced craving directly before the delivery of the intervention [48], it might well be that the delivery of an ABM intervention at home/online is more effective than for example the delivery in the laboratory or clinic in which substance use is unusual or even not allowed. All included studies of the current review that examined the effects of a home-delivered and/or online ABM intervention [23, 25, 28, 36] showed positive effects on symptoms of addiction, but given the inconclusive results with regard to baseline attentional bias and changes of attentional bias as well as other limitations of these studies (e.g. low study quality), it is yet unknown in which environment the delivery of ABM interventions is most effective. However, given the high accessibility and simple combination with other treatments, it seems reasonable to especially encourage more research into the effectiveness of home-delivered and online ABM interventions.

Second, another notable difference between studies is the amount of provided training sessions, varying from 1 to 21 sessions. As mentioned above, it seems that multiple sessions are necessary in order to achieve clinically meaningful effects. However, it is not yet clear how many sessions are needed to achieve a long lasting effect on attentional bias and substance use.

Third, in a similar vein, the duration of each training session varied across studies (i.e. 16 min to 1 h). Although the duration of the training sessions was often not reported, the diversity was also visible in the amount of provided trials, varying from 96 [31] to 960 [33] trials per training session. Future research might therefore not only want to investigate how many training sessions are necessary to achieve clinically relevant effects, but also how long each training session should be in order to be effective.

Fourth, the effectiveness of two different ABM interventions has been investigated—a training based on the dot probe paradigm (*n* = 15) and versions of the AACTP (*n* = 3). At first sight, the studies based on the AACTP intervention seem highly promising in terms of clinical relevance, as all three studies found significant positive effects on substance use-related symptoms. However, taking the small amount of studies and the fact that all three studies did not report on either baseline attentional bias or the modification of attentional bias into account, future research needs to verify the robustness of these positive findings. The findings with regard to the training based on the dot probe paradigm appeared to be quite unstable—seven studies found positive effects on changes of symptoms and eight did not find these effects. In line, several researchers from the field expressed concerns about the interpretation of results [43, 49] and the reliability [50, 51] of the dot probe task when used as an assessment tool. These concerns may also apply for the modified versions used as ABM interventions. Furthermore, the static character of the visual dot probe task may limit the generalisation to daily-life situations and therefore its positive effects on changes of symptoms. Future research might consider developing new ABM interventions that are more reliable and more realistic, to promote transfer of training effects to real-life situations.

Last, different outcome measures have been used to measure changes in substance use-related symptoms, and even when studies used the same outcome measure, it was often defined differently. This diversity makes not only the comparison of studies more challenging, but also limits the possibility of drawing conclusions about the effectiveness of ABM interventions in addiction. We encourage future research to include multiple outcome measures to further explore on which symptoms of addictive behaviour ABM interventions exert influence. The most important outcome measures might be the amount of used substance, craving, and relapse.

Furthermore, it was remarkable that only 3 of the 18 studies reported a power analysis, which was complied with a sufficient sample size. The other studies either missed to report a sufficient power analysis or did not reach the a priori set power level. The sample size of the studies varied from 40 to 434 participants, with a tendency for small samples. Given the possibility of underpowered studies, interpretations and conclusions based on the included studies should be considered with this limitation in mind.

Finally, we observed that changes of attentional bias were operationalised differently between studies. Whereas the majority of studies first investigated whether changes of attentional bias from baseline to post-test differed between groups (ABM group versus control group), several studies only examined whether attentional bias changed significantly within the ABM group from baseline to post-test. Given the possibility of a learning effect from baseline to post-test within both groups, changes of attentional bias might only be considered essential when these changes in the ABM group significantly differ from the changes in the active control group. Therefore, we suggest to not only examine changes within groups, but to also investigate the changes between groups over time.

### Limitations

Some comments are in order with regard to the limitations of the current review. First, although we carefully selected the most relevant databases and screened the articles, we cannot rule out that we might have missed a relevant study. Furthermore, the scope of included studies and the differences between the studies with regard to their design, type of substance use, and type of ABM intervention only allowed us to draw qualitative conclusions. Nevertheless, in our opinion, this systematic review contributed to the field of ABM interventions in addiction by synthesising current knowledge and by revealing important features of these studies that point to concrete recommendations and directions for future research. Finally, another limitation might be the large range in the quality of the studies that were included, with one study even scoring below the suggested cut-off score. Clearly, this implies that no firm conclusions can be drawn from the available evidence. Yet, at the same time, it points to the importance to identify this lack of quality as future research can benefit from it by improving their way of setting up the study and reporting on the results.

## Conclusion

Taken together, despite the inconsistency in findings, there are indications that multi-session ABM interventions might have clinically relevant effects on symptoms of addictive behaviour. This seems especially true in the case of alcohol. However, future research is needed to clarify the effectiveness of ABM interventions and should (i) report on both baseline attentional bias and attentional bias changes between groups over time, (ii) use different paradigms for the assessment task and the intervention to differentiate between direct learning effects and generalisation of new learned processes, (iii) include multiple outcome measures to further explore on which specific symptoms of addictive behaviour the effects of ABM interventions exert influence, (iv) use several follow-up measures over a long period of time, (v) investigate the efficacy of online and home-delivered ABM versus lab-delivered interventions, and (vi) include well-powered clinical samples.

## Additional files

Additional file 1: PRISMA checklist. (DOC 65 kb)

Additional file 2: Additional information on attentional bias calculation. (DOCX 13 kb)

Additional file 3: Overview of similarities/differences between included studies of the current systematic review and the meta-analysis by Cristea and colleagues [16]. (DOCX 13 kb)

## Abbreviations

ABM: Attentional bias modification
CBT: Cognitive behavioural therapy
AACTP: Alcohol Attention Control Training Programme
CBM: Cognitive bias modification
LEAP: Life Enhancement and Advancement Programme
TAU: Treatment as usual
