# Supplementary material for: The effectiveness of attentional bias modification for substance use disorder symptoms in adults: a systematic review
Source: Syst Rev. 2018 Oct 13;7:160. doi: 10.1186/s13643-018-0822-6 (PMC6186103; doi:10.1186/s13643-018-0822-6)
Supplement: Supplementary file 2 — Additional information on attentional bias calculation. (DOCX 13 kb) [file 13643_2018_822_MOESM2_ESM.docx]

**Additional file 2**

**Table 1** *T-tests based on table 2 in Lee & Lee (2015)*

|  | **Control group** | **ABM group** |
| --- | --- | --- |
| 0-200 ms | t(21) = 1.17; p = .255 | t(20) = 0.45; p = .658 |
| 200-400 ms | t(21) = 5.60; p < .001^a^ | t(20) = 3.70; p = .001^a^ |
| 400-600 ms | t(21) = 3.36; p = .003^a^ | t(20) = 2.71; p = .013^a^ |
| 600-800 ms | t(21) = -2.67; p = .014^a^ | t(20) = -1.90; p = .072 |
| 800-1000 ms | t(21) = -4.79; p < .001^a^ | t(20) = -4.26; p < .001^a^ |

^a^ Significant at p <.05.

**Table 2** *T-tests based on table 1 in Schoenmakers and colleagues (2007)*

| **ABM group** | t(53) = 0.59; p = .558 |
| --- | --- |
| **Control group** | t(53) = -0.62; p = .538 |

**Table 3** *T-tests based on figure 2 in Schoenmakers and colleagues (2010)*

| **ABM group** | t(20) = 0.48; p = .636 |
| --- | --- |
| **Control group** | t(21) = 0.78; p = .444 |

**Table 4** *T-tests based on figure 1 in Field and Eastwood (2005)*

| **Attend group** | t(19) = 1.35; p = .193 |
| --- | --- |
| **Avoid group** | t(19) = 1.31; p = .206 |
