# Supplementary material for: The effectiveness of attentional bias modification for substance use disorder symptoms in adults: a systematic review
Source: Syst Rev. 2018 Oct 13;7:160. doi: 10.1186/s13643-018-0822-6 (PMC6186103; doi:10.1186/s13643-018-0822-6)
Supplement: Supplementary file 3 — Overview of similarities/differences between included studies of the current systematic review and the meta-analysis by Cristea and colleagues [16]. (DOCX 13 kb) [file 13643_2018_822_MOESM3_ESM.docx]

**Additional file 3**

**Table 1** *Overview of similarities/differences between included studies of the current systematic review and the meta-analysis by Cristea and colleagues (2016)*

| **Authors, year** | **Current systematic review** | **Meta-analysis by Cristea and colleagues (2016)** | **Reason for difference** |
| --- | --- | --- | --- |
| Attwood et al., 2008 | X | X |  |
| Begh et al., 2015 | X | X |  |
| Charles et al., 2015 | X | - | Not included in meta-analysis because study is no randomized trial |
| Cox et al., 2015 | X | X |  |
| Elfeddali et al., 2016 | X | - | Published after last literature search of meta-analysis in December 2015 |
| Fadardi & Cox, 2009 | X | - | Not included in meta-analysis because study is no randomized trial |
| Field & Eastwood, 2005 | X | X |  |
| Field et al., 2007 | X | X |  |
| Field et al., 2009 | - | X | Excluded from the current review because study’s inclusion criteria not in line with current inclusion criteria of systematic review |
| Kerst & Waters, 2014 | X | X |  |
| Lee & Lee, 2015 | X | - | Not clear why study is not included in meta-analysis as it was online available March 2015 and is a randomized trial in problem drinkers |
| Lopes et al., 2014 | X | X |  |
| Mayer et al., 2016 | X | - | Published after last literature search of meta-analysis in December 2015 |
| McGeary et al., 2014 | X | X |  |
| McHugh et al., 2010 | X | X |  |
| Schoenmakers et al., 2007 | X | X |  |
| Schoenmakers et al., 2010 | X | X |  |
| Wiers et al., 2015 | X | X |  |
| Ziaee et al., 2016 | X | - | Published after last literature search of meta-analysis in December 2015 |

*Legend.* A cross (i.e., X) indicates that this study is included in the paper, whereas a stripe (i.e., -) indicates that this study was not included.
